# Description of a multicenter safety checklist for intraoperative hemorrhage control while clamped during robotic partial nephrectomy

**DOI:** 10.1186/1754-9493-6-8

**Published:** 2012-04-02

**Authors:** Kenneth G Nepple, Gurdarshan S Sandhu, Craig G Rogers, Mohamad E Allaf, Jihad H Kaouk, Robert S Figenshau, Michael D Stifelman, Sam B Bhayani

**Affiliations:** 1 Washington University School of Medicine, St. Louis, MO, USA; 2 Henry Ford Hospital, Detroit, MI, USA; 3 Johns Hopkins University, Baltimore, MD, USA; 4 Cleveland Clinic, Cleveland, OH, USA; 5 New York University School of Medicine, New York, NY, USA; 6 Department of Surgery, Division of Urology, Washington University School of Medicine, 660 S. Euclid Avenue, St Louis, MO 63110, USA

**Keywords:** Kidney neoplasms, Robotics, Nephrectomy, Hemorrhage, Patient safety

## Abstract

**Background:**

The adoption of robotic assistance has contributed to the increased utilization of partial nephrectomy for the management of renal tumors. However, partial nephrectomy can be technically challenging because of intraoperative hemorrhage, which limits the ability to identify the tumor margin and may necessitate the conversion to open surgery or radical nephrectomy. To our knowledge, a comprehensive safety checklist does not exist to guide surgeons on the management of hemorrhage during robotic partial nephrectomy. We developed such an safety checklist based on the cumulative experiences of high volume robotic surgeons.

**Methods:**

A treatment safety checklist for the management of hemorrhage during robotic partial nephrectomy was collaboratively developed based on prior experiences with intraoperative hemorrhage during robotic partial nephrectomy.

**Results:**

Reducing the risk of hemorrhage during robotic partial nephrectomy begins with reviewing the preoperative imaging for renal vasculature and tumor anatomy, with a focus on accessory vessels and renal tumor proximity to the renal hilum. During hilar exposure, an attempt is made to identify additional accessory renal arteries. The decision is then made on whether to clamp the hilum (artery +/- vein). If bleeding is encountered during resection, management is based on whether the bleeding is suspected to be arterial or from venous backbleeding. Operative maneuvers that may increase the chance of success are highlighted in safety checklists for arterial and venous bleeding.

**Conclusions:**

Safely performing robotic partial nephrectomy is dependent on attention to prevention of hemorrhage and rapid response to the challenge of intraoperative bleeding. Preparation is essential for maximizing the chance of success during robotic partial nephrectomy.

## Introduction

Hemorrhage during surgery is a patient safety concern and a source of stress for surgeons. During partial nephrectomy, in which part of the kidney is removed for a renal tumor, hemorrhage can be particularly troublesome because the kidney is a well perfused organ and renal cell carcinomas are associated with increased vascularity [1]. Traditionally, partial nephrectomy had been performed via an open approach but there has been a steady trend toward a minimally invasive approach [2].

As partial nephrectomy is increasingly performed with a minimally invasive approach, control of intraoperative hemorrhage can be more challenging than simple manual compression of the renal parenchyma with suturing, as can be done in open surgery. Hemorrhage is further concerning as control can occupy valuable time during which the renal unit is ischemic due to renal vascular clamping during tumor excision. The surgeon is essentially "on the clock" to unclamp the renal unit as soon as possible to prevent acute tubular necrosis and further loss of renal function. During this stressful situation, it is imperative for the surgeon to have an organized approach to management of hemorrhage, as delayed management could lead to prolonged ischemic times, iatrogenic positive surgical margins from inadequate visualization, conversion to open surgery, or radical nephrectomy.

Series on robotic partial nephrectomy report a reasonable blood loss (Table 1). Nevertheless, the specific situation of "bleeding while clamped" is likely underreported. Experts who publish their results may seamlessly manage this issue without adverse events, and thereby avoid complications in their published series. Thus, the existing literature does little to educate surgeons on how to deal with bleeding during robotic partial nephrectomy while clamped.

**Table 1:** Selected reports from 2009-2012 of estimated blood loss during robotic partial nephrectomy with hilar clamping.

Reference	Year	Series size	Estimated blood loss (mean or median)	Estimated blood loss (range)
**Ficarra et al **[3]	2012	347	100 (median)	50-150 (IQR)
**Castillo et al**[4]	2012	25	440 (mean)	20-2000 (range)
**Dulabon et al**[5]	2011	446	262 for hilar vs. 208 for nonhilar (p = 0.14) (mean)	50-1250 for hilar, 0-2200 for nonhilar (range)
**Naeem et al**[6]	2011	97	150 for obese vs. 100 for nonobese (p = 0.03) (mean)	75-250 for obese, 50-150 for nonobese (IQR)
**Kaouk et al**[7]	2011	252	332 for initial vs. 248 for contemporary (p = 0.04) (mean)	50-1400 for initial, 10-2200 for contemporary (range)
**Lorenzo et al**[8]	2011	65	243 (mean)	0-1600 (range)
**Williams et al**[9]	2011	27	180 (mean)	NA
**Petros et al**[10]	2011	95	150 if prior surgery vs. 100 if no prior surgery (p = 0.14) (mean)	69-250 if prior surgery, 50-200 if no prior surgery (IQR)
**Gong et al**[11]	2010	29	220 (mean)	100-370 (range)
**Patel et al**[12]	2010	71	100 for ≤ 4 cm vs. 100 for > 4 cm (p = 0.29) (median)	75-200 for ≤ 4 cm vs. 50-200 for > 4 cm (IQR)
**Benway et al**[13]	2010	183	132 (mean)	10-900 (range)
**Scoll et al**[14]	2010	100	127 (mean)	50-800 (range)
**Benway et al**[15]	2009	50	140 (mean)	25-450 (range)
**Benway et al**[16]	2009	129	155 (mean)	NA
**Ho et al**[17]	2009	20	189 (mean)	50-260 (range)

Adapted from Benway et al.[18] (IQR = Interquartile range, NA = Not available)

The goal of this manuscript is to educate robotic surgeons in factors to minimize hemorrhage during robotic partial nephrectomy. We identified factors to consider during review of preoperative imaging and renal hilar exposure. We then developed a safety and management checklist for "hemorrhage while clamped" during robotic partial nephrectomy.

## Methods

Robotic partial nephrectomy was initially reported in 2004 [19]. Since that time, the authors have performed a total of over 1500 robotic partial nephrectomies. During educational courses on robotic urological surgery from 2007 to 2010, it was noted that comments were made regarding management of hemorrhage during robotic partial nephrectomy. Suggestions for management were made from audience members, experts, and other attendees of the courses. However, starting in 2011, there were no new suggestions or options, suggesting "saturation" of management options. Based on these comments, a safety checklist with the mentioned options for managing hemorrhage was constructed for educational purposes by two of the authors (KN and SB) and then revised based on comments from the multi-institutional group of authors.

## Results

### Preoperative imaging

Avoiding hemorrhage during robotic partial nephrectomy begins with review of the preoperative imaging, which for renal tumors typically includes CT or MRI with contrast. The imaging should be reviewed carefully, in axial and coronal planes, for the presence of accessory renal arteries which may be identified in greater than 20% of cases [20]. Accessory renal arteries become important because if such an artery is not occluded during hilar clamping, then substantial arterial bleeding can occur during tumor resection. Tumor size and vicinity to renal hilar vessels is also inspected on preoperative imaging, as larger and more central tumors have an increased risk of hemorrhage [21].

### Renal hilar dissection and clamping

Prior to renal tumor dissection, the renal hilum (renal artery and vein) is exposed. Surgical dissection should proceed slowly to avoid inadvertent vascular injury and blood loss during this portion of the procedure, and it is useful to have vascular suture (i.e. 4-0 Prolene on an RB-1 needle anchored with a LAPRA-TY clip) available for vascular suturing if necessary to control venous bleeding. An accessory renal artery is sometimes identified which was not visualized on preoperative imaging. Further delineation of renal arterial anatomy (to identify accessory renal arteries or confirm adequate clamping) may be assisted by the use of a laparoscopic Doppler ultrasound probe [22] or near infrared fluorescence of intravenous indocyanine green [23]. Even in cases where there is only one renal artery, the surgeon must be mindful of clamping the renal artery after it has already branched. If arterial clamping is performed too distally, an unoccluded proximal renal artery branch supplying the tumor itself can result in significant arterial bleeding during resection.

In the case of tumors of increased complexity (hilar tumors, endophytic, larger tumors), additional dissection can be considered to improve hilar exposure. Additional dissection to consider include: extra dissection of the renal hilar vessels proximally and distally, ligation of the gonadal vein to improve exposure, or dissection of the adrenal gland away from the kidney. These measures may improve hilar access if needed for subsequent clamping with a laparoscopic Satinsky clamp or use of a stapler if conversion to radical nephrectomy is required.

Once the hilar anatomy is delineated and the tumor is exposed, the decision is then made on how to perform hilar clamping. Available methods of hilar clamping include assistant-placed laparoscopic vascular clamps, robotic surgeon-placed clamps, or placement of a laparoscopic Satinsky clamp. The correct choice of clamp is based primarily on surgeon preference. If the Satinsky clamp is placed then an additional assistant port must be placed and care must be taken to avoid a collision between a robotic arm and the clamp, which could potentially lead to avulsion of the hilum and significant hemorrhage.

Following renal hilar and tumor exposure, a decision is then made to clamp the artery alone or to clamp both the artery and the vein. For small, exophytic renal tumors, clamping of the artery alone is typically sufficient. However, for tumors that are more endophytic, the possibility of encountering a sizable venous branch during excision is increased and therefore clamping of the renal vein should also be considered.

### Bleeding source during tumor resection: arterial versus venous

All suggestions for intraoperative management of hemorrhage during robotic partial nephrectomy involved identification if the hemorrhage was arterial or venous in origin, as management options differed depending on this critical branch point. Several clues may help the surgeon identify whether the bleeding source is arterial or venous. Arterial bleeding is pulsatile and typically of a larger volume while venous bleeding may generally be more of an ooze and of a lower volume. In additional to the initial assessment of bleeding as arterial versus venous, communication should also occur with anesthesia to alert them to the possibility of hemodynamic instability from hemorrhage so that appropriate support with intravenous fluid and blood transfusion may be given as needed.

### Arterial bleeding

**Figure 1:**
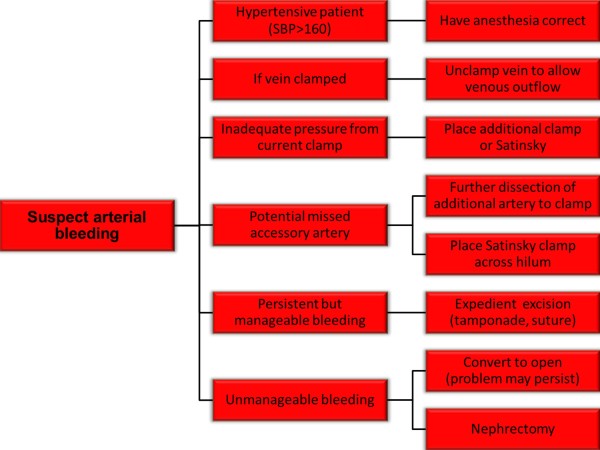
**Proposed safety checklist for the management of arterial bleeding during robotic partial nephrectomy**.

The arterial safety checklist (Figure 1) addresses management of arterial bleeding from the partial nephrectomy bed. Often more than one of these maneuvers may be needed. Two suggestions involve adjunct measures, which despite not directly controlling the arterial bleeding may nonetheless help to decrease the amount of hemorrhage and allow for the opportunity to identify the arterial source of bleeding. First, if the patient is hypertensive, then controlled reduction of blood pressure by anesthesia may improve visualization. A second temporizing option is to unclamp the renal vein. This option at times may substantially improve visualization by allowing venous outflow in the setting of continued arterial inflow into the partial nephrectomy bed.

Further suggestions for the management of arterial bleeding focus on methods to control the actual renal arterial branch which is the source of the bleeding. These include placement of an additional clamp for arterial control, either on the same artery if there is inadequate clamp pressure or on an accessory artery if one is identified with further dissection.

Even with the aforementioned efforts, arterial bleeding may persist. If this bleeding persists at a manageable level, then excision of the renal mass can continue expediently with consideration of adjunct measures to control bleeding such as tamponade of the bleeding (by placement of pressure by the surgical assistant with suction device or other instrument) or by placement of a suture at the resection bed even if the mass has only been partially excised. Once the mass is excised then further hemostatic measures can be performed including cauterizing the base of the resection bed, suturing the resection bed, and placing sliding clip renorrhaphy sutures [15] ultimately providing compression of the parenchyma. However, if bleeding is not manageable, then importantly, emergent open conversion (for open partial nephrectomy or complete nephrectomy) or robotic nephrectomy may be necessary.

### Venous backbleeding

**Figure 2:**
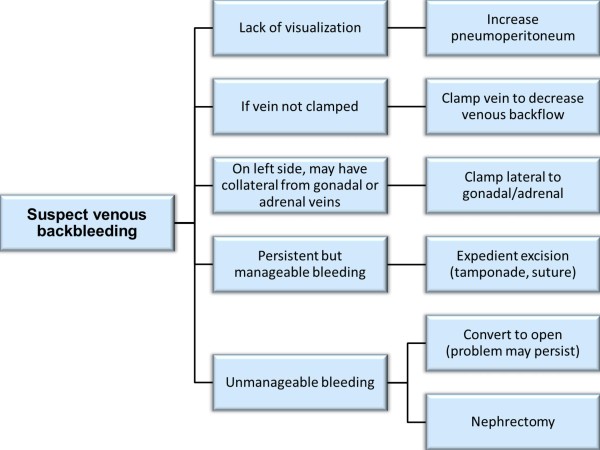
**Proposed safety checklist for the management of venous backbleeding during robotic partial nephrectomy**.

The management safety checklist of venous hemorrhage (Figure 2) is generally technically easier to manage than arterial bleeding. The insufflation pressure maintaining the pneumoperitoneum can be increased up to 18 mm Hg to improve visualization. This measure is typically well tolerated by most patients, although anesthesia should be informed of the need to monitor for increases in end tidal carbon dioxide or difficulty with ventilation. The theoretical increased risk of gas embolism with increased pneumoperitoneum pressures has been hypothesized [24], but the authors are not aware of the actual occurrence [25].

In contrast to vein unclamping with arterial bleeding, with venous bleeding an additional clamp can be placed on the renal vein to decrease venous backbleeding. Additionally, on the left side placement of the clamp on the renal vein lateral to the gonadal and adrenal veins may stop additional backbleeding from those branches. In practice, the tumor can often be excised despite venous hemorrhage which can subsequently be controlled with suturing the tumor bed. Conversion to open surgery for radical nephrectomy for venous bleeding during minimally invasive partial nephrectomy is uncommon [26,27].

## Discussion

Hemorrhage during robotic partial nephrectomy is probably underreported, as most series are reported by high volume surgeons at tertiary care centers who may not have major consequences from this bleeding. Therefore, hemorrhage may not result in a formal complication if it is quickly and efficiently controlled. Nevertheless, as hemorrhage may occur during critical and often stressful portions of the operation, an array of management options should be recognized by the surgeon performing the procedure. We present a comprehensive discussion of factors to consider regarding hemorrhage during robotic partial nephrectomy. It is our hope that review of these factors will educate other surgeons in preparation for this experience, and thus increases patient safety and surgeon confidence in dealing with this difficult situation.

In practice, hemorrhage prevention may be more important that hemorrhage management. Hemorrhage can be prevented quite simply with accurate identification of all arteries and veins, and use of adequately functioning vascular clamps. Unfortunately, such a statement presumes that the surgeon ability, radiographic imaging, and patient anatomy are uniform, easily identified, and subject to no variability. In "real world" situations, all of our high volume experts have dealt with hemorrhage during partial nephrectomy while clamped and thus have contributed to the discussion of options.

The safety checklists present an array of options, however good clinical judgment remains paramount. For example, a surgeon may not improve hemostasis by implementing measures for venous hemorrhage in a patient that actually has arterial hemorrhage instead. Additionally, as the brisk bleeding with arterial hemorrhage can be difficult to manage and can result in rapid patient instability, the decision to eventually convert to an open procedure or radical nephrectomy to allow for safe completion of the surgery is ultimately based on sound clinical acumen which guides the surgeon through their organized approach to such a scenario.

On an educational and safety level, knowledge of the potential options for hemorrhage management may prevent worsening of conditions, particularly amongst lower volume surgeons. Although testing such safety checklists is impossible, discussion of the management of hemorrhage is important to disseminate in the literature in an effort to potentially improve patient safety.

## Conclusions

Safely performing robotic partial nephrectomy is dependent on attention to prevention of hemorrhage and rapid response to the challenge of intraoperative bleeding. Preparation is essential for maximizing the chance of success during robotic partial nephrectomy.

## Competing interests

The authors declare that they have no competing interests.

## Authors' contributions

KN and SB conceived and drafted the manuscript. All authors participated in critical revision of the manuscript. All authors read and approved the final manuscript.

## References

[B1] Vira MA Novakovic KR Pinto PA Linehan WM Genetic basis of kidney cancer: a model for developing molecular-targeted therapies BJU Int 2007 99 1223 1229 10.1111/j.1464-410X.2007.06814.x 17441915

[B2] Dulabon LM Lowrance WT Russo P Huang WC Trends in renal tumor surgery delivery within the United States Cancer 2010 116 2316 2321 20225227 10.1002/cncr.24965 PMC4235157

[B3] Ficarra V Bhayani S Porter J Buffi N Lee R Cestari A Mottrie A Predictors of warm ischemia time and perioperative complications in a multicenter, international series of robot-assisted partial nephrectomy Eur Urol 2012 61 395 402 10.1016/j.eururo.2011.10.046 22079308

[B4] Castillo OA Rodriguez-Carlin A Lopez-Fontana G Vidal-Mora I Gomez IR Robotic Partial nephrectomy: An initial experience in 25 consecutive cases Actas Urol Esp 2012 36 15 20 21831484 10.1016/j.acuro.2011.06.003

[B5] Dulabon LM Kaouk JH Haber GP Berkman DS Rogers CG Petros F Bhayani SB Stifelman MD Multi-institutional analysis of robotic partial nephrectomy for hilar versus nonhilar lesions in 446 consecutive cases Eur Urol 2011 59 325 330 10.1016/j.eururo.2010.11.017 21144643

[B6] Naeem N Petros F Sukumar S Patel M Bhandari A Kaul S Menon M Rogers C Robot-assisted partial nephrectomy in obese patients J Endourol 2011 25 101 105 10.1089/end.2010.0272 21204675

[B7] Kaouk JH Hillyer SP Autorino R Haber GP Gao T Altunrende F Khanna R Spana G White MA Laydner H 252 robotic partial nephrectomies: evolving renorrhaphy technique and surgical outcomes at a single institution Urology 2011 78 1338 1344 10.1016/j.urology.2011.08.007 22001098

[B8] Lorenzo EI Jeong W Oh CK Chung BH Choi YD Rha KH Robotics applied in laparoscopic kidney surgery: the Yonsei University experience of 127 cases Urology 2011 77 114 118 10.1016/j.urology.2010.02.011 20434761

[B9] Williams SB Kacker R Alemozaffar M Francisco IS Mechaber J Wagner AA Robotic partial nephrectomy versus laparoscopic partial nephrectomy: a single laparoscopic trained surgeon's experience in the development of a robotic partial nephrectomy program World journal of urology 2011 DOI: 10.1007/s00345-011-0648-5 10.1007/s00345-011-0648-5 21274541

[B10] Petros FG Patel MN Kheterpal E Siddiqui S Ross J Bhandari A Diaz M Menon M Rogers CG Robotic partial nephrectomy in the setting of prior abdominal surgery BJU Int 2011 108 413 419 10.1111/j.1464-410X.2010.09803.x 21176077

[B11] Gong Y Du C Josephson DY Wilson TG Nelson R Four-arm robotic partial nephrectomy for complex renal cell carcinoma World J Urol 2010 28 111 115 10.1007/s00345-009-0427-8 19499225

[B12] Patel MN Krane LS Bhandari A Laungani RG Shrivastava A Siddiqui SA Menon M Rogers CG Robotic partial nephrectomy for renal tumors larger than 4 cm Eur Urol 2010 57 310 316 10.1016/j.eururo.2009.11.024 19945213

[B13] Benway BM Bhayani SB Rogers CG Porter JR Buffi NM Figenshau RS Mottrie A Robot-assisted partial nephrectomy: an international experience Eur Urol 2010 57 815 820 10.1016/j.eururo.2010.01.011 20116163

[B14] Scoll BJ Uzzo RG Chen DY Boorjian SA Kutikov A Manley BJ Viterbo R Robot-assisted partial nephrectomy: a large single-institutional experience Urology 2010 75 1328 1334 10.1016/j.urology.2009.10.040 20080290 PMC2879432

[B15] Benway BM Wang AJ Cabello JM Bhayani SB Robotic partial nephrectomy with sliding-clip renorrhaphy: technique and outcomes Eur Urol 2009 55 592 599 10.1016/j.eururo.2008.12.028 19144457

[B16] Benway BM Bhayani SB Rogers CG Dulabon LM Patel MN Lipkin M Wang AJ Stifelman MD Robot assisted partial nephrectomy versus laparoscopic partial nephrectomy for renal tumors: a multi-institutional analysis of perioperative outcomes J Urol 2009 182 866 872 10.1016/j.juro.2009.05.037 19616229

[B17] Ho H Schwentner C Neururer R Steiner H Bartsch G Peschel R Robotic-assisted laparoscopic partial nephrectomy: surgical technique and clinical outcomes at 1 year BJU Int 2009 103 663 668 10.1111/j.1464-410X.2008.08060.x 18990172

[B18] Benway BM Bhayani SB Surgical outcomes of robot-assisted partial nephrectomy BJU Int 2011 108 955 961 10.1111/j.1464-410X.2011.10470.x 21917097

[B19] Gettman MT Blute ML Chow GK Neururer R Bartsch G Peschel R Robotic-assisted laparoscopic partial nephrectomy: technique and initial clinical experience with DaVinci robotic system Urology 2004 64 914 918 10.1016/j.urology.2004.06.049 15533477

[B20] Ramadan SU Yigit H Gokharman D Tuncbilek I Dolgun NA Kosar P Kosar U Can renal dimensions and the main renal artery diameter indicate the presence of an accessory renal artery? A 64-slice CT study Diagn Interv Radiol 2011 17 266 271 20698003 10.4261/1305-3825.DIR.3507-10.0

[B21] Simhan J Smaldone MC Tsai KJ Canter DJ Li T Kutikov A Viterbo R Chen DY Greenberg RE Uzzo RG Objective measures of renal mass anatomic complexity predict rates of major complications following partial nephrectomy Eur Urol 2011 60 724 730 10.1016/j.eururo.2011.05.030 21621910 PMC3319121

[B22] Hyams ES Perlmutter M Stifelman MD A prospective evaluation of the utility of laparoscopic Doppler technology during minimally invasive partial nephrectomy Urology 2011 77 617 620 10.1016/j.urology.2010.05.011 21109296

[B23] Tobis S Knopf J Silvers C Yao J Rashid H Wu G Golijanin D Near infrared fluorescence imaging with robotic assisted laparoscopic partial nephrectomy: initial clinical experience for renal cortical tumors J Urol 2011 186 47 52 10.1016/j.juro.2011.02.2701 21571337

[B24] Neudecker J Sauerland S Neugebauer E Bergamaschi R Bonjer HJ Cuschieri A Fuchs KH Jacobi C Jansen FW Koivusalo AM The European Association for Endoscopic Surgery clinical practice guideline on the pneumoperitoneum for laparoscopic surgery Surg Endosc 2002 16 1121 1143 10.1007/s00464-001-9166-7 12015619

[B25] Weld KJ Ames CD Landman J Morrissey K Connor T Hruby G Allaf ME Bhayani SB Evaluation of intra-abdominal pressures and gas embolism during laparoscopic partial nephrectomy in a porcine model J Urol 2005 174 1457 1459 10.1097/01.ju.0000173010.96639.85 16145470

[B26] Richstone L Seideman C Baldinger L Permpongkosol S Jarrett TW Su LM Pavlovich C Kavoussi LR Conversion during laparoscopic surgery: frequency, indications and risk factors J Urol 2008 180 855 859 10.1016/j.juro.2008.05.026 18635228

[B27] Galvin DJ Savage CJ Adamy A Kaag M O'Brien MF Kallingal G Russo P Intraoperative conversion from partial to radical nephrectomy at a single institution from 2003 to 2008 J Urol 2011 185 1204 1209 10.1016/j.juro.2010.11.077 21334022

